# Imagining a post-antibiotic era: a cultural analysis of crisis and antibiotic resistance

**DOI:** 10.1136/medhum-2022-012409

**Published:** 2022-08-03

**Authors:** Kristofer Hansson, Adam Brenthel

**Affiliations:** 1 Department of Social Work, Malmö University, Malmö, Sweden; 2 Division of Art History and Visual Studies, Department of Arts and Cultural Sciences, Lund University, Lund, Sweden

**Keywords:** Infectious diseases, Medical humanities, internet, medical anthropology

## Abstract

The study presented in this article is about the role played by imagination when national and international organisations convey the idea of a dystopian crisis involved in the real transition to a postantibiotic era. The present is an era that can be defined as a time when no new antibiotics are discovered or developed, and existing antibiotics simultaneously become less effective since bacteria develop resistance against the active substances. Today, antibiotic resistance is an international fact; thousands of people die every year in Europe and the USA as a result of bacteria that have become resistant. Then, imagination can conjure up a different and a much more dystopian future. This article stems from a public debate concerning the global increase of antibiotic resistance; and will examine how the concept of fantasy and imagination is central in picturing such a future crisis in society. The article’s empirical basis mainly consists of reports from global and Swedish organisations, dating from the 1990s and onwards. These fantasies show that our society has a strong urge to always try to understand and explain present time and to identify how ‘our’ era relates to the past as well as the future. The concept of crisis plays an important role in these fantasies, it is key to use it when thinking about change. The analysis builds on texts and illustrations from global organisations like the WHO and also national authorities in Sweden that aim to convey the science behind the challenge. The aim is to develop a theoretical and empirical understanding, from the perspective of cultural analysis, of how fantasy and crisis are linked when the future is conceived.

## Introduction

This article addresses the role played by imagination in how national and international organisations convey the idea of a dystopian crisis to describe the transition to a postantibiotic era; this is a new era in which bacteria have become resistant to existing antibiotics and the antibiotics no longer work. With the help of imagination, an image can be visualised that moves human thoughts into the future—the far future or a future close at hand. An idea is thereby created of how people in this future might handle, or not handle, such medical crises when infections that may turn into sepsis, or advanced stages of pneumonia. Other researchers have pointed out how historical experiences are used to create visions of this future (Brown 2019; Brown and Nettleton 2017). This article stems from a public debate, mainly in Sweden and UK, concerning the global increase of antibiotic resistance; and will examine how the concept of fantasy and imagination is central in picturing such a future crisis in society. This study finds that the discussion consists of a mixture of well-founded empirical knowledge and dramatic fictionalisation of such knowledge (cf. Brenthel and Hansson 2017). The article particularly focuses on the discussions that describe and explain the growing resistance to antibiotics as a period when humanity enters what is termed as the postantibiotic era. The analysis builds on texts and illustrations from global organisations like the WHO and also national authorities that aim to convey the science behind the challenge. The present is an era that can be defined as a time when no new antibiotics are discovered or developed, and existing antibiotics simultaneously become less effective since bacteria develop resistance against the active substances and can therefore be seen as a time between how it was and how it is going to be.

Today, antibiotic resistance is an international fact; thousands of people die every year in Europe and the USA as a result of bacteria that have become resistant. Then, imagination can conjure up a different and a much more dystopian future. Thus, an estimate is that in 2050, 10 million people will die of infections that cannot be treated because of resistant bacteria and ineffective antibiotics (AMR-review 2014). Imagination can be regarded as a possibility of travelling in thought to a dystopian society deeply struck by such a manifest crisis. The idea that our global society is moving into the postantibiotic era seems to have started in the early 1990s. This is when the first texts with the conclusion that the current increasing resistance and absence of new antibiotics would form human history in times to come were published (Cohen 1992). The history of antibiotic resistance is older than that; it was an obvious fact for Alexander Fleming even when antibiotics started to be produced for public benefit in the beginning of the 1940s. In his Nobel lecture in 1945, he speculated about whether the bacteria might adapt to the new antibiotic world (Fleming 1945). The aim of this article is to develop a theoretical and empirical understanding, from the perspective of cultural analysis, of how fantasy and crisis are linked when the future is conceived.

### Background: a dystopian fantasy requiring action to be taken

The article begins with an example of how descriptions of the future can be used in various contexts to depict a national or a global situation in crisis. Based on the ideas of ethnologist Sjöstedt Landén, fantasy is regarded as a theoretical concept that encompasses everything, possible and impossible, that a person can imagine (Sjöstedt Landén 2011; cf. Glynos 2010; Scott 2011). The main point of the analysis is therefore not to study what is true or what is false, but rather to examine the connotations about the future that are created in the writing of the fantasy. The first example will analyse not only how the fantasy is told, but also the significance of the visual context for the setting of the fantasy.

Sometimes, few words are needed to create a fantasy of the future. The former UK Prime Minister David Cameron was interviewed in 2014 by the British Broadcasting Corporation about a newly published review that explained why so few antimicrobial drugs had been introduced during the past years (Fergus 2014). While presenting the review, Cameron’s intention in this interview was to show his power of action by introducing the panel of experts from science, finance, industry and global health, led by the economist Jim O'Neill, who were now trying to solve the problem. From the title of the review the content can be discerned, ‘Antimicrobial Resistance: Tackling a crisis for the health and wealth of nations’ (AMR-review 2014). There was a crisis coming up that must be prevented by powerful political action so as not to risk the health and welfare of nations. In an almost historical show, Cameron used the TV interview to evoke a fantasy among the viewers of what to expect in a future when antibiotics no longer have any effect. In the interview he expressed himself in a way that has later been quoted numerous times in various forms of media: ‘If we fail to act, we are looking at an almost unthinkable scenario where antibiotics no longer work and we are cast back into the Dark Ages of medicine’. While the quote gives the viewers an immediate picture of the future; at the same time it can be criticised for being too apocalyptic (Brown and Nettleton 2017).

**Figure 1:**
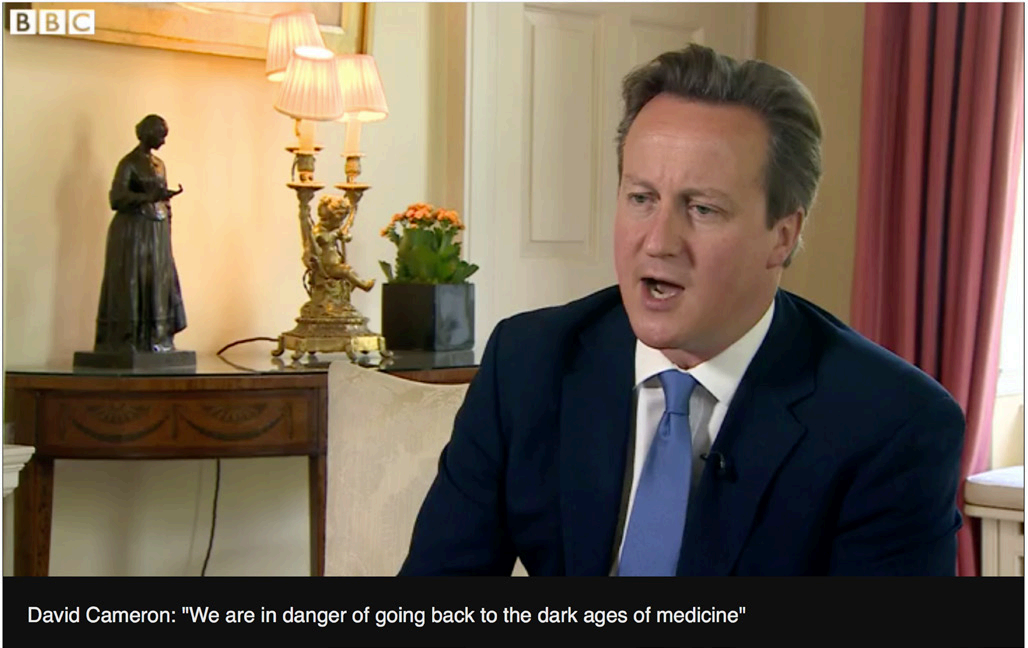
The picture is from the British Broadcasting Corporation (BBC interview with Prime Minister David Cameron in 2014; it shows a traditional interview situation with a prominent politician (Fergus 2014). This clip from the interview also clearly illuminates how confidence is created visually through choice of clothes, physical appearance and background. In this case, he is wearing an elegant and discreet jacket, white shirt and tie; he is well combed and newly shaved. The background also establishes him in the context of a privileged man.

This brief but forceful sentence directly catches the viewers’—at least those living in a society with access to antibiotics—attention, evoking a fantasy of a possible future based on a constructed depiction of the past—The Dark Ages. Society risks not only being arrested in its development but ‘cast’ back to a time Cameron metaphorically calls ‘the Dark Ages of medicine’. This arouses the imagination of a time that most of us have experienced through books we have read, or popular science films we have seen. Perhaps our mind’s eye conjures up people being bled or having their skulls bored into, perhaps limbs that were so infected that they had to be sawn off under severe pain. It is also a fantasy that creates images, which originate from many Hollywood films about the horror of the uncontrollable.1 Of course, it is impossible to know exactly what Cameron is referring to, but his statement works well in its political context. It is interesting to conduct a closer study of the context in which this statement was uttered, in order to analyse the form of ideology that this fantasy about the future connotes. In the TV interview, the camera angle features Cameron from the waist up, showing a traditional interview situation with a prominent politician (Fergus 2014). He is neatly groomed and wearing clothes that signal his position of power, namely a white shirt, a tie and an elegant and discreet jacket; he is well combed and newly shaved. Further, the interview is conducted in an environment that cannot be defined as either his home or his workplace. In the background is an older table lamp, a plant and an art statue on a table. In this way the background establishes him in the context of a privileged man. Visually, there is thus nothing that relates to the fantasy he is depicting. Instead, the material setting acts to promote confidence in the political action he is proposing in the interview. This is made clear in the introduction; Cameron says (figure 1):

I think this is a very serious threat. We are in danger of going back to the Dark Ages of medicine. To see infections that were treatable, not be treatable, and we will see many thousands of people potentially die from these infections. So, it is a very, very serious problem. And one we absolutely have to grip. We have to grip it globally, because this is a problem that will affect every country in the world and Britain is providing leadership to make that happen. (Fergus 2014)

The ‘unthinkable scenario’ of our society is here set into the context of Cameron and Britain taking the political responsibility for this impending ‘crisis for the health and wealth of nations’. To visualise and draw public attention to this crisis, Cameron uses fantasy not only to compel the viewers to imagine the future, but he also presents the viewers with a very specific problem. The result of this crisis is said to be that many thousands of people across the world risk dying prematurely because antibiotics have lost their effect. Fantasy is used in this context to call for political change and to promote the actions that Cameron is now taking responsibility for by appointing a panel of experts who will handle the question. Simultaneously, such statements fire apocalyptic fantasies that the world is transforming into something new and dangerous; a development that cannot be stopped. The return to an old world is suddenly inescapable and the modern belief in progress appears totally lost. The political meaning of this loss is part of a broader change and plays into this fantasy.

As the example above shows, fantasy is a central point in drawing attention to the impending crises that was envisioned by Cameron, thus requiring political resolution and action to be taken by society. From Cameron’s point of view, it was a case of clarifying that he was a politician who shouldered the responsibility for the situation. It also seems as if he was defining himself and his country as the driving force in taking on the task of saving the world from a global crisis caused by the resistance of bacteria to antibiotics. Thus, the fantasy in conjunction with the political message acted in a normalising way to induce the public to accept that Cameron assumed the responsibility for the situation (cf. Howarth 2010). However, this also becomes a political statement that is not only intended to grip hold of people; the grip can also be found to extend beyond the actual scope of facts that were known when the statement was uttered (cf. Glynos 2010).

In order to understand the way in which people’s imagination is invoked, this line of reasoning can be associated with a perception of crises that is linked, not with the actual circumstances, but with plausible future events. This concept of crisis has a long history (Koselleck 1982).2 Such crises may be financial, personal or societal, but the common points of the perspective include the actual describing of the problem that has arisen and that earlier approaches to the facts have needed to be revaluated. This means that the course of the crisis involves a time before, during and after the crisis. This can also be understood with a focus of risk in late modernity, and how science today has a central role in society when research makes these crises visible before they happened. As sociologist Beck notes, risk becomes known through knowledge production in science (Beck 1986). It is knowledge that will play a central role in the risk assessments that, for example, the politicians need to make to reduce risk factors. But it is not only science based on knowledge; fantasies are also central to created narratives about an impending crisis. Antibiotic resistance *might be* at this stage currently, being a risk factor to drive the development of resistant bacteria. Another point is that the concept of crisis is regularly used in a historical sense to reconstruct a past course of events concerning where and when the crisis arose and when society changed course (Magnusson Staaf 2013).

Our perspective of fantasy and crisis is connected with the Swedish philosopher Gustafsson’s concept of the privilege of formulating the problem (Gustafsson 1989). This reasoning draws attention to the circumstance that those who have the privilege of defining the problem are also in command of proposing the solution. Another aspect linked with this privilege is what Gustafsson terms the information privilege; this means that those who have the knowledge and information can usually use this to define the problem. Such a privilege to formulate the problem is included in research that illuminates invisible risks that surround us in modern society; increasing resistance to antibiotics can be regarded as this kind of risk (Beck 1986). Scientific knowledge can be used to formulate the problem, and thus define which problems exist concerning antibiotic resistance, and how to distribute resources to solve the problem. Or in other words, how to avoid the crisis. Thus, Cameron uses his privilege to formulate the problem by showing the economic effects of handling the resistance to antibiotics. This has been analysed by Brown and Nettleton who write that for Cameron:

[…] the biotic has become the promissory medium for ‘economic imaginary’ performing modes of imagining and projecting visions of the proper workings of economy. This might include the discourse of ‘free choice’ exercised by patients newly positioned as consumers, or re-engineering the market motivations of the pharmaceutical industries (Brown and Nettleton 2017: 495).

The privilege to formulate the problem will not, for example, concern the global solidarity of providing all people of the earth with healthcare, nutritious food and sanitary facilities. The purpose of this example is to stress that Beck’s reasoning about the role of science in society—of creating an information privilege—does not sufficiently explain the privilege to formulate the problem; what is needed is imagination (Beck 1986). With the help of imagination, politicians like Cameron can grip a hold of the public and show them where and how the problem will be solved. Power in this case is not only intertwined in the clothes he wears and the context in which he is interviewed, nor in the scientific facts he can present—*his privilege of being informed*. Power is also closely connected with how well he conveys a fantasy about the dystopian crisis awaiting us—read the Western world—in future if we do not act now (Caduff 2015). Here, it does not mean whether or not it is realistic, but rather if it is a scenario that people easily can imagine, catch onto and adjust their actions accordingly. On the other hand, it might be difficult to imagine and therefore might not become part of peoples’ consciousness. Maybe it is in line with our thoughts and ideas about the future: certainly, there are many films about epidemics and outbreaks that are based on real events or future threats. These threats can be seen as central both in the politicians’, as well as organisations’, risk assessments processes, at the same time as a narrative about a dystopian crisis that is awaiting (Beck 1986). Consequently, what we are interested in is to conduct a closer study of reports that are produced by organisations and to find out how fantasy can be used to understand these reports. In the next section we will address this question.

### The impending crisis

‘The Dark Ages of medicine’ is a narrative that Cameron creates to appeal to the general ideas of the public about ancient medicine. This type of narrative can be found in many contemporary international reports illuminating the situation of antibiotic resistance (cf. Brenthel and Hansson 2017; Hansson, Lenander, and Loodin 2021). The article will here provide further examples to show how these narratives are constructed and to illuminate the fantasies that emerge.

In the same year as Cameron’s report was issued, the WHO reported in ‘*Antimicrobial Resistance: Global Report on Surveillance 2014*’ that humanity had been in a ‘discovery void’ since 1987 (WHO 2014). This referred to the fact that virtually no new antibiotics had been introduced since that year. Medicines marketed as new after 1987 were instead variations or combinations of already known substances. Thus, the report was pointing out the same problem that was brought to light in the review ‘*Antimicrobial Resistance: Tackling a Crisis for the Health and Wealth of Nations*’ (AMR-review 2014). In the WHO’s report, a temporality occurs that can be said to have a good deal of similarities with Cameron’s ‘the Dark Ages of medicine’, which we will analyse here, in order to illustrate the way in which the fantasy of the impending crisis requires a course of events.

**Figure 2:**
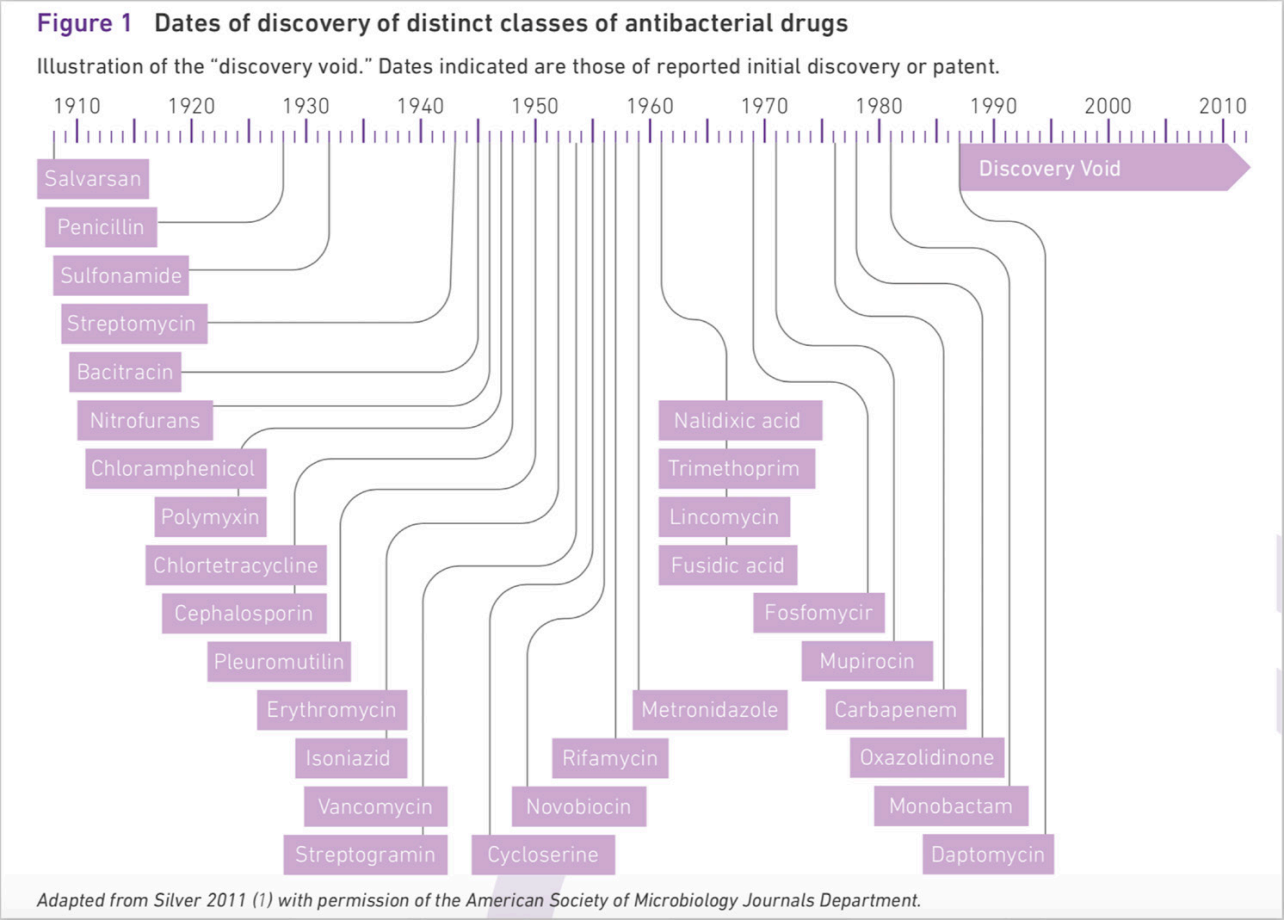
The diagram (Silver 2011, adapted with permission from WHO (WHO 2014)) illustrates the discussion in the chapter ‘Resistance to antibacterial drugs’. In the introduction of the chapter, it is stressed that ‘[t]he development of resistance is a normal evolutionary process for microorganisms, but it is accelerated by the selective pressure exerted by widespread use of antibacterial drugs‘ (WHO 2014: 1). The diagram was used to visualise how easy it was to pick the low-hanging fruit represented by the first antibiotics. The further on in time, the fewer discoveries, until only the void opens. The void is deep and the result is lost hope; the fantasy of continuous discovery of better antibiotics died and was replaced by a world without antibiotics. Both visions are exaggerated, like fantasies always are; this is part of the logics of fantasy.

In figure 2, the WHO report visualises the timeline from the first discovery of antibiotics in the 1920s to the discovery of the last class in 1986. On this timeline, the discoveries of the various antibiotics are marked, but from 1987 onwards there is an arrow with the text ‘Discovery Void’, running to 2010 and beyond. The diagram illustrated that a multitude of different types of penicillin were produced during the so-called medical golden age around the 1950s. This golden age is placed in the middle of the illustration in such a way, it becomes the central period in the diagram. When we arrive at the 1970s and 1980s, discoveries start to thin out; we can only find two new discoveries in the latter decade in the diagram. There are no more after 1987, instead the ‘Discovery Void’ unfolds. This period is marked by a thick arrow distinctly pointing towards the future; a future that is not shown in the diagram: what might occur afterwards is left to our imagination.3 The temporality is based on three different dimensions; the first dimension contains twentieth-century history and medical success. Then comes a dimension that can be said to focus on the present time or the near future. No development has occurred in this dimension, and science has started to discern risks that will arise when no new antibiotics are discovered to fight the bacteria that have developed resistance against the antibiotics presented in the first stage. The third dimension is the future. While this dimension is based on the two former dimensions, we still do not know what will happen; only fantasy and imagination can evoke ideas of the future.

However, the visualisation of the ‘Discovery Void’ is not the only point that reveals the use of fantasy to illustrate an impending crisis. Even the phrase ‘Discovery Void’ is a strong metaphor in this context; the lack of new antibiotics to be discovered is equated with a void—emptiness. The empty space becomes a metaphor for an apocalyptic fantasy in which humanity is lost and can no longer find its way. This spatial metaphor helps us to think and relate to the abstract future and the invisible risk. The metaphoric use of space to visualise the effects of a crisis also occurs in other crisis narratives. A common way to present a crisis is to describe society or the involved parties as lost, groping round in empty space, trying to find a solution that will take them out of this space or room (Hansson 2013). As pointed out by Bachelard, spaces or rooms in a house are part of our way of thinking and relating to the world around us (Bachelard 2014).

Other dystopian images in the form of metaphors can be used to illustrate the contemporary situation and future developments. For instance, in a massive online open course lecture at Uppsala University, the phrase ‘the silent tsunami‘ was used as a metaphor for antibiotic resistance (Uppsala University 2017). In the description of the course, it was stated that more strains of bacteria are developing resistance against existing medicines at the same time as ‘the pipeline of new antibiotics is now almost dry’. The world is flooded with dangerous bacteria while the pipeline delivering new medicine is almost dry; two metaphors are juxtaposed, the flood wave and the trickle. Almost all people in Sweden have a relation to a tsunami nowadays after the earthquake in the Indian Ocean in 2004, where many people suffered and 543 people from Sweden lost their lives. In this metaphor, the threat is something that suddenly overwhelms us with no previous warning, and without being able to defend ourselves. We also know from the tsunami in 2004 that it came silently; it was not expected and in an instant the flood wave washed over everything, ravaging all things in its way. When these two metaphors are used in relation to antibiotic resistance, an image is created of us suddenly being struck by powers so strong that we cannot resist them, we are completely washed over.

**Figure 3:**
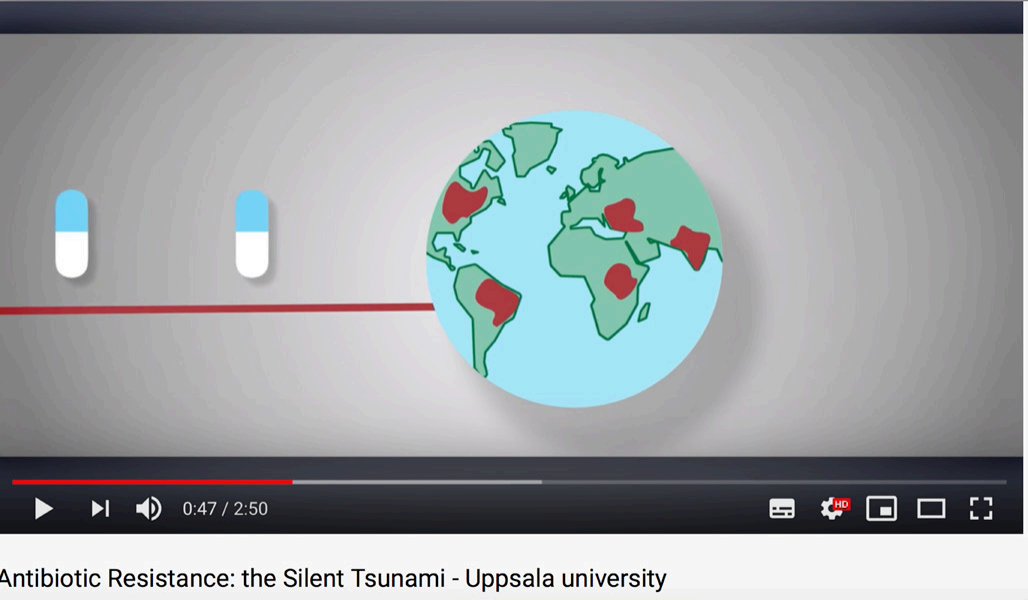
This YouTube video (ReActTube) shows a highly illustrative and educational information film about the discovery of penicillin and all the good this medicine has brought, and how it has gradually and unnoticeably changed bacteria which we live with (ReActTube 2017). The result is a greater number of deaths and the contemporary situation of these alarming red patches showing where resistant bacteria are developing. ReAct, Action on Antibiotic Resistance.

The network ReAct (Action on Antibiotic Resistance)4 produced a YouTube video in 2017 to disseminate information about a similar issue that can be seen in figure 3 (ReActTube 2017). Like the temporality in the previously analysed report, the narrative of this video is based on the temporality of bacteria developing resistance over time. Furthermore, in the video, the temporality is contextualised at a global level. In the video a timeline—a red line—visualises the development of resistant bacteria, and then red patches gradually spread across the surface of the earth to indicate where the problem of antimicrobial resistance is increasing. This is the slowly growing ‘silent tsunami’.

Such metaphors are not unusual, for example, when scientists write about the body and its relation to bacteria and medicine. Cultural conceptions about humans and microbiota have a long history, illustrating that humans are continuously generating dichotomous thinking about bacteria and the body. In her book *Flexible Bodies*, published in 1995, anthropologist Martin maintains that advancement of science in the 1950s and 1960s led to cultural conceptions about bacteria as opposed to the human body (Martin 1994). Martin points out that bacteria became domesticated as either good or bad for humans; this development was largely an effect of medical advancements after World War II. Dangerous bacteria had to be handled and controlled as a health-promoting measure to benefit the population. It was a development that was founded in changing views in the nineteenth century when authorities began to take responsibility for the well-being of the population. This perspective was strengthened in what has been called ‘The Golden Age of Medicine’ after World War II. However, as Martin points out, modern research on bacteria has changed this view and today there is a development that accentuates symbiosis between humans and healthy bacteria. Instead of regarding the body as a fortress that needs to be defended against dangerous bacteria, a flexible body that needs healthy bacteria is now brought to the foreground. Medical science kept dangerous bacteria under control by applying effective antibiotics. Nevertheless, metaphors do not act independently. Rather, when metaphors such as these are used in the debate, they are associated with societal developments said to involve the entering of a new era.

### The postantibiotic era

An early forerunner to the idea of bacteria resistance as a future era rather than a contemporary problem can be found in 1992 in Cohen’s article ‘Epidemiology of Drug Resistance: Implications of a Post-Antimicrobial Era’ (Cohen 1992). This was only 6 years after the last antibiotic to be discovered was launched on the market according to the visual in figure 2). In the introduction, Cohen depicts a hospital ward in an age he terms as the preantimicrobial era. The patients in the beds are not suffering from cancer or heart diseases, but from illnesses such as pneumonia and syphilis. In the antimicrobial era, the difficulties of these illnesses, or the ‘memories of the pre-antimicrobial era’ (Cohen 1992: 1050) in the words of Cohen, were rapidly forgotten. He elaborates on this reasoning:

The emergence of multi-drug resistance in *Mycobacterium tuberculosis*, *Streptococcus pneumoniae*, *Staphylococcus aureus*, *Enterococcus*, *Shigella dysenteriae*, and *Plasmodium falciparum* has made many currently available antimicrobial drugs ineffective and in certain instances is already posing important public health problems. Furthermore, scientists at a recent National Institute of Health workshop reported that fewer new antimicrobial drugs were under development. Such issues have raised the concern that we may be approaching the post-antimicrobial era. (Cohen 1992: 1050)

The introduction of the article, together with the subtitle ‘Implications of a Post-Antimicrobial Era’, captures the previously mentioned temporality, and uses the dangers of the first era to depict a fantasy of the implications of this for the postantimicrobial era. In this way, Cohen illustrates the risks that began to be discerned in the early 1990s, but it is our, the readers, imagination that helps us understand the crisis that will arise if we do not take the risks seriously. A main point is that young people will die prematurely, which Cohen stresses is what happened in the preantimicrobial era. ‘Many of the patients were young, and most would die of the disease or its complications’ (Cohen 1992: 1050). In such a way, Cohen creates a vision of the risk of humanity entering an entirely new era and illuminates what this era would entail.

To speak in terms of an era connotes something more far-reaching than a crisis; it implies fundamental transformation of society as we know it. The transition from the current era into the imagined era may undoubtedly be described as a crisis, although humanity is not necessarily in crisis once the new era has become established. It is interesting to compare the postantimicrobial era with the more recent idea of the Anthropocene Period, introduced by Crutzen in 2007 (Palsson *et al*. 2013). The intention of this concept is to highlight that the total human impact on earth is as great as the geological powers that formed the earth. The future world will be formed by human activity; these activities leave traces in geological layers and will be detectable in the future. Like the postantibiotic era, the Anthropocene Period is a new and revolutionary idea that evokes a feeling that the world is heading towards an unknown state, when previously favourable conditions for human development are threatened, or thrown into disarray. Furthermore, it is not possible to turn back. The idea of the Anthropocene Period can, in this way, help us to understand the link between actual processes in nature and biology, and the fantasies that seems to be needed to understand these processes and their consequences. It is as if the fantasies are needed to categorise between a then and a now, or a near future, to define a line that has been crossed.

A further few articles from the 1990s discuss the problem of resistance from the point of view that this era has already arrived or is soon to be expected, and that the crossed line is a fact. One is Hamilton-Miller’s article ‘Living in the “Post-Antibiotic Era”’ (Hamilton-Miller 1997), published in 1997, where he clarifies the problem.

The progressive increase in bacterial antibiotic resistance has reached such a degree that there is now talk of ‘the end of the antibiotic era’. While new chemical entities that act directly on pathogens, such as the oxazolidinones and everninomicin, and a variety of different types of peptide antibiotics are very welcome and may provide a short-term solution, this stereotyped response is not the answer to the overall problem. It is clear that other strategies to combat the spread and effects of microbial resistance are needed. (Hamilton-Miller 1997: 2)

Here, temporality is specifically mentioned; humanity is considered to have come to the end of ‘the antibiotic era’ and we must now start to reflect on how we should tackle the new era. The WHO report, ‘Antimicrobial Resistance: Global Report on Surveillance’, published in 2014, revitalised the idea of a ‘post-antibiotic era’ in media. The report states that antibiotic resistance is a ‘problem so serious that it threatens the achievements of modern medicine’ (WHO 2014: IX). This is in agreement with the fantasies previously described. WHO calls for commitment concerning the question, in order to avoid the postantibiotic era, which would mean that we have not yet entered this era. This is clarified in the statement, ‘[w]ithout urgent action we are heading for a post-antibiotic era, in which common infections and minor injuries can once again kill‘ (WHO 2017). The concept of the postantibiotic era is used as a comprehensive fantasy into which a number of horrendous images can be incorporated. Some of the fantasies created by the use of this era belong to a discourse of fear.

### Discourse of fear

**Figure 4:**
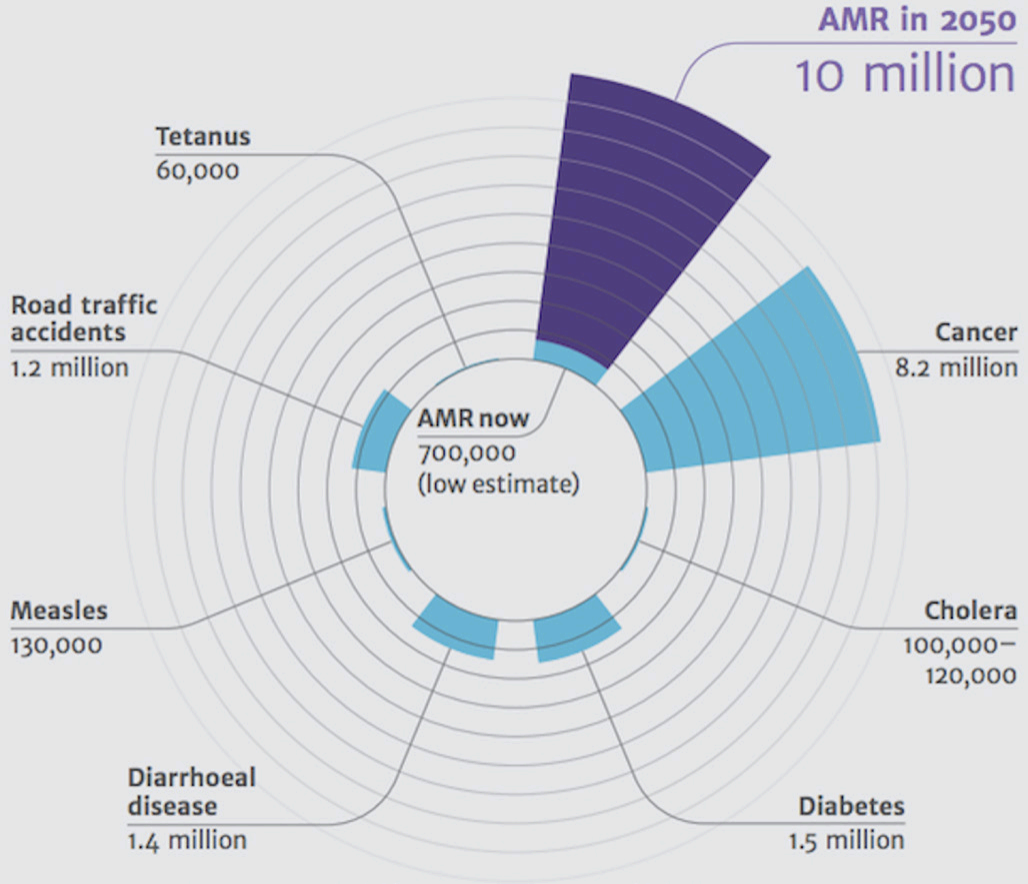
A figure from the report ‘Antimicrobial Resistance: Tackling a crisis for the health and wealth of nations’ showing the number of deaths caused by antimicrobial resistance (AMR) now and in the future, in 2050, set in relation to other common diseases and accidents (AMR-review 2014). The rose chart is an effective way to develop understanding through comparison. In addition, the creators of this chart have succeeded in including a dimension of time, showing the expected development of AMR until 2050, if no action is taken.

When studying official documents—in the form of reports from organisations, interviews with persons in a position of power, YouTube videos from international networks and so on—it is striking that there are so few photographs showing the consequences of resistant bacteria. We have to watch documentary films, or rather Hollywood films, to find such visualisations. A vivid example is seen in the previously quoted review that Cameron talked about: ‘Antimicrobial Resistance: Tackling a Crisis for the Health and Wealth of Nations’ (AMR-review 2014). In this report the consequences of not addressing antimicrobial resistance are visualised in a rose chart (see figure 4). The diagram illustrates an alarming future in which there will be 10 million deaths in 2050 if no drastic measures are taken. This is compared with the 8.2 million people who currently die of cancer. Other diseases and accidents are included on the chart to further the point, including: diabetes 1.5 million people, road traffic accidents 1.2 million people and cholera 100 000–120 000. In this review analysis, this visualisation of death is a way of creating a discourse of fear, inducing us, the readers, to understand the severity of the situation. The apocalyptic fantasy becomes concrete through figures, although there is a lack of photographs in this kind of publication. Photographs that often do occur show either multicoloured medicines of various kinds, or Petri dishes with bacteria cultures. These are, moreover, aesthetically attractive pictures showing exciting shapes and beautiful colours.

This discourse of fear is accentuated even in the text (Altheide 2002). The introduction of the report contains the language: ‘A post-antibiotic era – in which common infections and minor injuries can kill – far from being an apocalyptic fantasy, is instead a very real possibility for the 21st century’ (WHO 2014: IX). Wording—such as ‘apocalyptic fantasy’—that does not necessarily have connotations to the real world is used here; it is a kind of wording we would find in a film. In the next follow-up report ‘Tackling drug-resistant infections globally: Final report and recommendations’ (AMR-review 2016), this becomes even more obvious.

The magnitude of the problem is now accepted. We estimate that by 2050, 10 million lives a year and a cumulative 100 trillion USD of economic output are at risk due to the rise of drug-resistant infections if we do not find proactive solutions now to slow down the rise of drug resistance. Even today, 700 000 people die of resistant infections every year. Antibiotics are a special category of antimicrobial drugs that underpin modern medicine as we know it: if they lose their effectiveness, key medical procedures (such as gut surgery, caesarean sections, joint replacements, and treatments that depress the immune system, such as chemotherapy for cancer) could become too dangerous to perform. (AMR-review 2016: 4)

To frighten people to attention, the concept of the postantibiotic era is used as a fantasy for the future. This form of alarmism has been analysed by sociologist Nerlich; she identifies a postantibiotic apocalypse with similarities to the current way of treating societal challenges such as climate change and terrorism (Nerlich 2009). This is a discourse of fear that media, for example, easily can resort to when communicating news about measures taken by organisations and authorities to impede the threat (Altheide 2002). In a popular science journal published by Gothenburg University, the journalist employs this kind of discourse to attract interest for the research performed at the university. In the introduction, it is mentioned that ‘not being able to rely on effective antibiotics in the future would involve a catastrophic situation. No operation or even childbirth could be performed without severe risk. We would in effect be back in the Middle Ages’ (Carina 2015). In this case, the phrase ‘the Dark Ages of medicine’ is transferred—or mixed up!—to the Middle Ages, making the concept even more frightening, since the Middle Ages is often associated with diseases such as leprosy, plague and the black death.

**Figure 5:**
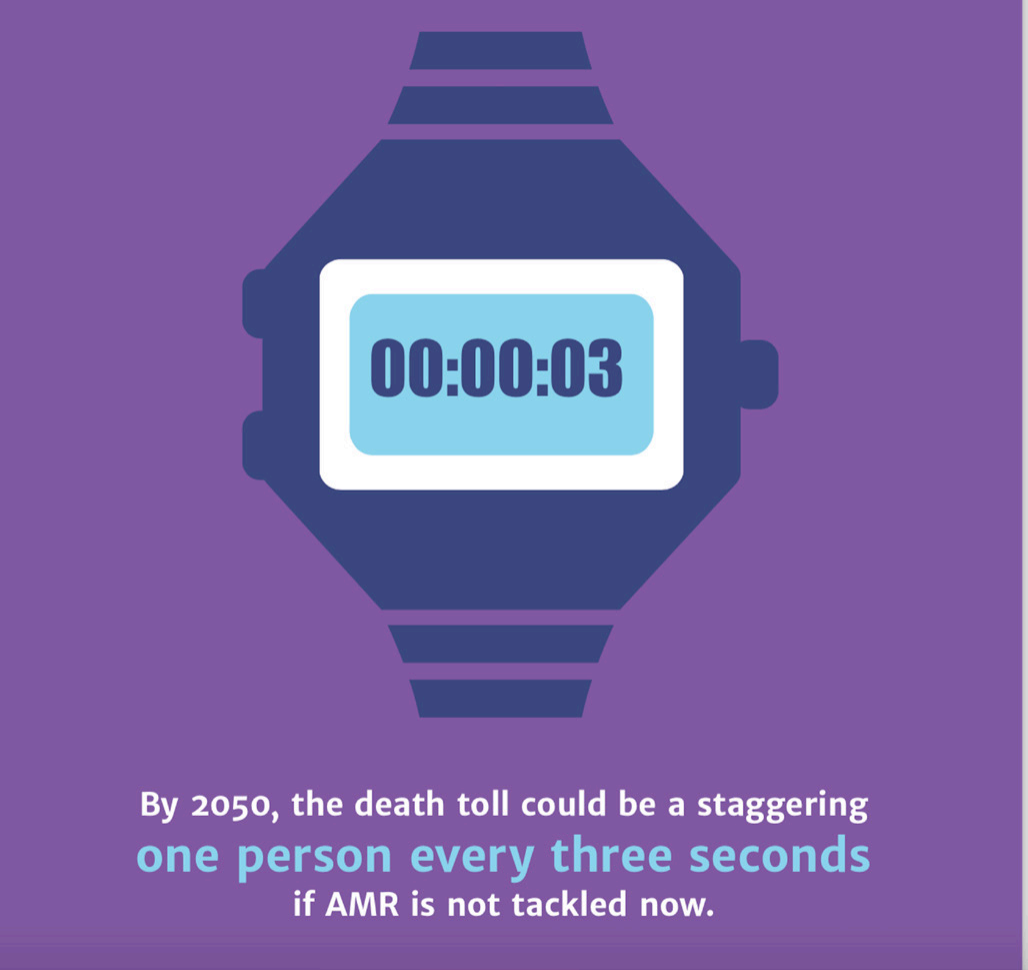
This rather visual digital watch can be seen in the report ‘Tackling drug-resistant infections globally: Final report and recommendations’ (AMR-review 2016). The purpose is to clarify that society must act forcefully now, in order to avoid a future crisis when one person might die every third second due to resistant bacteria. This illustration can be found in the latter part of the report under the heading ‘If not tackled, rising AMR could have devastating impact’ (AMR-review 2016). The page thus has many similarities with an advertisement enticing us to consume products and services. AMR, antimicrobial resistance.

A central point in this discourse of fear is that when it is presented by an authority, there is an undertone of this authority having the situation under control, knowing what to do or having already taken these important measures. In a section found late in the report ‘Tackling drug-resistant infections globally: Final report and recommendations’ (AMR-review 2016), a visualisation such as this can be found; it is a discourse of fear and of the impending crisis, although it conveys hope at the same time (see figure 5). It shows a digital watch with the digit 3 in the secondhand field, illustrating the risk of one person dying every third second if we cannot solve the problem of resistant bacteria. Under the clockit says: ‘By 2050, the death toll could be a staggering one person every three seconds if AMR is not tackled now’. This illustration looks almost like an advertisement, but it calls for a reaction: society must act now and must not wait for the future and the crisis that is near. It puts hope in focus!

### The hope of avoiding the crisis

Even though the discourse of fear is in full force in the fantasies about the postantibiotic era, there is also hope that this is a problem that can be solved. This hope obviously resides in the nature of the privilege of formulating the problem; the involved parties who have the power to define the problem, are also able to come up with a solution, which is often a case of how to distribute financial resources. Consequently, there was nothing strange in the fact that an economist—O’Neill—was assigned the task of leading the British review, to return to the article’s initial discussion (AMR-review 2014). The 2014 review ends on a hopeful note with the following statement under the heading ‘This crisis can be averted if the world takes action soon’ (AMR-review 2014: 15).

This might be one of the world’s biggest problems, but it does not need to be its hardest.

At the core of this Review is the conviction that we need to preserve and further support the huge progress in medicine and poverty alleviation that has taken place over the last 25 years. It would be unforgiveable if the great progress made in combatting infectious diseases could be threatened by the lack of new drugs that are within reach, or for lack of common sense investment in infrastructure that keeps us safe from avoidable infections. (AMR-review 2014: 15)

Previous research within social science has shown that the discourse of hope is central, for example, in medical research that recurrently evokes hopeful fantasies about future cures (Brown and Michael 2003). The research can be said to be oriented towards the future, with the perspective that any looming problems are probably possible to solve if enough resources are forthcoming from research funds. Hope exists as a realistic expectation (Hyun 2013). Hope is therefore closely associated with the fantasy of the crisis; hope makes it possible to imagine a solution that will prevent the crisis. The relationship between hope and crisis is pivotal in the discussion about the postantibiotic era, not least for organisations and authorities with the responsibility of formulating the problem and providing solutions. In a Swedish context, responsible politicians, among others, can be seen to use this kind of argumentation.

Sweden is often described as a leading country, at least by Swedish politicians and researchers. Social Democrat Gabriel Wikström was Cabinet Minister of the Ministry of Health and Social Affairs in 2014–2017, responsible for the questions of public health, medical treatment and sports. He states on the website of the Swedish Government concerning the course of action taken against antibiotic resistance, ‘Sweden is successful in the work against antibiotic resistance, but we must not permit ourselves to become less vigilant. This is not a battle we can win by ourselves – when the bacteria spread across the world, we must all unite in finding solutions’ (Gabriel 2016). Such a hopeful strategy was, for example, issued by the Swedish Government Offices in 2016 in ‘Swedish Preparatory Work against Antibiotic Resistance’, which was signed by no less than three ministers in charge (Regeringskansliet 2016). The following is stated in the introduction.

Sincere commitment and forceful action against antibiotic resistance is necessary if future generations are to be able to trust in effective cures for our most common infections even in times to come. Antibiotics must be safe-guarded as a communal and valuable resource providing all people who need it the benefits offered by effective antibiotics. (Regeringskansliet 2016: 3)

In this case, we do not find the discourse of fear. Instead, we see politicians signalling that they are ready to take action with a clear plan and a number of objectives for the work of averting the crisis. In the same way the former UK Prime Minister David Cameron did in 2014, when he took action to solve the problem with antibiotic resistance.

## Discussion

As soon as we realise how dependent healthcare is on antibiotics today, the idea of being without anyseems like a nightmare, an apocalyptic crisis. The ‘the post-antibiotic era’ thus evokes the feeling of reaching the far end of our current era, very similar with the Anthropocene Period. In our view, establishing our current modern situation as the ‘antibiotic era’, the prefix post arouses ideas of a future when there are no longer any antibiotics. We have noted that there are remarkably few concrete pictures or illustrations of this impending era (cf. Brenthel and Hansson 2017; Hansson, Lenander, and Loodin 2021). Rather, distinct ideas about what might happen are created through descriptions in words. Indeed, the lack of pictures does not mean that we have difficulties in imagining such circumstances. On the contrary, imagination appears to be of central significance when authorities and organisations outline this threatening future as a way to mobilise political and economic power for proposing a solution (Gustafsson 1989).

However, this might just be a productive prospect that can be applied in our specific current postantibiotic era. What we mean is that we are now in a situation when the idea of antibiotics as a ‘magic bullet’ is already a remedy of the past, since bacteria have in fact developed various degrees of resistance against most antibiotics. We are beginning to become aware that we appear to be losing the battle, but we still have not reached the new dystopian state. As soon as society has reached the postantibiotic era, fantasies no longer produce a productive prospect. In that case, we will have reached a problematic reality and other fantasies will most likely come into existence. The purpose of these contemporary fantasies about a postantibiotic era is thus to avoid such a new looming reality.

Crises and fantasy enable a closer study of how global organisations and authorities act in relation to risks that are not necessarily visible to us, although they create a feeling of threat or fear. This might be applicable to resistant bacteria—not visible to the eye—or it might apply to global warming. An important point is how this preknowledge, mentioned by Beck, becomes a significant tool to visualise the problems that occur in society and how they are to be solved (Beck 1986). However, this does not seem to be sufficient; imagination and fantasy must also be included in the privilege of formulating the problem in order to form and clarify a future when the problems have become a reality and the crisis is a fact (Gustafsson 1989).

Fantasies that prevail today primarily concern the postantibiotic era and what this might mean for humanity, even if there are many real cases that could be categorised as postantibiotic. These fantasies show that our society—read the Western world—has a strong urge to always try to understand and explain the present time and to identify how ‘our’ era relates to the past as well as the future. The concept of crisis plays an important role in these fantasies; it is key to use it when thinking about change, largely because we have, throughout history, regarded the occurrence of crises as way to explain the downfall of earlier societies and the rise of new ages. This narrative tradition of ours seems to be so strong that we use it in our contemporary society to tell stories about our own times that always seem to bring us nearer a crisis that will take place in times to come in our imagined future.

## Data Availability

Data sharing is not applicable as no data sets were generated and/or analysed for this study.

## Notes

1. A few examples are: *Contagion* (2011, Steven Soderbergh), *I am legend* (2007, Francis Lawrence), *Pandemic* (2016, John Suits), *Resistance* (2015, Michael Graziano) and *Outbreak* (1995, Wolfgang Petersen).

2. The study’s purpose here is to point out the different connotations of the concept, which is implicated in showing how narratives are created to define when in the course of events a crisis arose or may come to arise.

3. The diagram is used to visualise how easy it was to ‘pick the low-hanging fruit’ represented by the first antibiotics. The further on in time, the fewer discoveries, until only the void opens. The void is deep and the result is lost hope; the fantasy of continuous discovery of better antibiotics died and was replaced by a world without antibiotics. Both visions are exaggerated, like fantasies always are; this is part of the logics of fantasy.

4. ReAct was created in 2005 and is an international independent network that articulates antibiotic resistance and its drivers. (17 March 2020; https://www.reactgroup.org/)

## References

[R1] Altheide D. L . 2002. Creating Fear: News and the Construction of Crisis. New York: Aldine de Gruyter.

[R2] AMR-review . 2014. “Antimicrobial Resistance: Tackling a Crisis for the Health and Wealth of Nations. London: The Review on Antimicrobial Resistance.” https://amr-review.org/sites/default/files/AMR%20Review%20Paper%20-%20Tackling%20a%20crisis%20for%20the%20health%20and%20wealth%20of%20nations_1.pdf.

[R3] AMR-review . 2016. “Antimicrobial Resistance: Tackling Drug-Resistant Infections Globally: Final Report and Recommendations. London: The Review on Antimicrobial Resistance.” https://amr-review.org/sites/default/files/160525_Final%20paper_with%20cover.pdf.

[R4] Bachelard G . 2014. The Poetics of Space. London: Penguin Classics.

[R5] Beck U . 1986. Risik o Gesellschaft: Auf Dem Weg in Eine Andere Moderne. Suhrkamp: Frankfurt am Main.

[R6] Brenthel A. , and Hansson K. . 2017. “Den Postantibiotiska Eran. Kulturella Perspektiv På Antibiotikaresistens.” Working Papers in Medical Humanities 3 (1): 1–41.

[R7] Brown N . 2019. Immunitary Life. London: Palgrave Macmillan. 10.1057/978-1-137-55247-1.

[R8] Brown N. , and Michael M. . 2003. “A Sociology of Expectations: Retrospecting Prospects and Prospecting Retrospects.” Technology Analysis & Strategic Management 15 (1): 3–18. 10.1080/0953732032000046024.

[R9] Brown N. , and Nettleton S. . 2017. “‘There Is Worse to Come’: The Biopolitics of Traumatism in Antimicrobial Resistance (AMR).” The Sociological Review 65 (3): 493–508. 10.1111/1467-954X.12446.

[R10] Caduff C . 2015. The Pandemic Perhaps. Oakland: University of California Press. 10.1525/9780520959767.

[R12] Carina E . 2015. “‘Antibiotikaresistens – Ett Hot Mot Mänskligheten.’ Science Faculty Magazine.” https://sciencefacultymagazine.se/okategoriserad/antibiotikaresistens-ett-hot-mot-manskligheten/.

[R11] Cohen M. L . 1992. “Epidemiology of Drug Resistance: Implications for a Post-Antimicrobial Era.” Science (New York, N.Y.) 257 (5073): 1050–55. 10.1126/science.257.5073.1050. 1509255

[R34] Fergus W . 2014. “‘Antibiotic Resistance: Cameron Warns of Medical “Dark Ages”.’ BBC.” www.bbc.com/news/health-28098838.

[R13] Fleming A . 1945. “Penicillin. Nobel Lecture, December 11.”

[R33] Gabriel W . 2016. “”Ny Strategi För Att Bekämpa Antibiotikaresistens.” Stockholm: Regeringskansliet.” http://www.regeringen.se/pressmeddelanden/2016/04/ny-strategi-for-att-bekampa-antibiotikaresistens/Film.

[R14] Glynos J . 2010. “The Grip of Ideology: A Lacanian Approach to the Theory of Ideology.” Journal of Political Ideologies 6 (2): 191–214. 10.1080/13569310120053858.

[R15] Gustafsson L . 1989. Problemformuleringsprivilegiet: Samhällsfilosofiska Studier. Stockholm: Norstedt.

[R16] Hamilton-Miller J. M. T . 1997. “Living in the ‘Post-Antibiotic Era’: Could the Use of Probiotics Be an Effective Strategy?” Clinical Microbiology and Infection 3 (1): 2–3. 10.1111/j.1469-0691.1997.tb00242.x. 11864067

[R17] Hansson K . 2013. “De Svåra Kriserna i Lifvet Kringgår Man Inte’: Psykiska Kriser Och Synen På Den Utvecklande Människan.” In Kris Och Kultur. Kulturvetenskapliga Perspektiv På Kunskap, Estetik Och Historia, edited by Mats Arvidson , Ursula Geisler , and Kristofer Hansson . Lund: Sekel Bokförlag.

[R18] Hansson K. , Lenander C. , and Loodin H. . 2021. Att Leva Med Bakterier: Möjligheter till Ett Levbart Immunitärt Liv. Lund: Pufendorfinstitutet, Lunds universitet.

[R19] Howarth D . 2010. “Power, Discourse, and Policy: Articulating a Hegemony Approach to Critical Policy Studies.” Critical Policy Studies 3 (3–4): 309–35. 10.1080/19460171003619725.

[R20] Hyun I . 2013. “Therapeutic Hope, Spiritual Distress, and the Problem of Stem Cell Tourism.” Cell Stem Cell 12 (5): S1934-5909(13)00146-X: 505–7. 10.1016/j.stem.2013.04.010. 23642359

[R21] Koselleck R . 1982. “Krise.” In Geschichtliche Grundbegriffe: Geschichtliches Lexikon Zur Politisch-Sozialen Sprache in Deutschland, edited by Werner Conze and Koselleck Reinhart . Stuttgart: Klett-Cotta.

[R22] Magnusson Staaf B . 2013. “Att Lita På Krisen? Om Kris Som Komponent i Historieteori.” In Kris Och Kultur: Kulturvetenskapliga Perspektiv På Kunskap, Estetik Och Historia, edited by Mats Arvidsson , Ursula Geisler , and Hansson Hansson . Lund: Sekel Bokförlag.

[R23] Martin E . 1994. Flexible Bodies: Tracking Immunity in American Culture from the Days of Polio to the Age of AIDS. Boston: Beacon Press.

[R24] Nerlich B . 2009. “‘The Post-Antibiotic Apocalypse’ and the ‘War on Superbugs’: Catastrophe Discourse in Microbiology, Its Rhetorical Form and Political Function.” Public Understanding of Science (Bristol, England) 18 (5): 574–88. 10.1177/0963662507087974. 20027773

[R25] Palsson G. , Szerszynski B. , Sörlin S. , Marks J. , Avril B. , Crumley C. , Hackmann H. , et al . 2013. “Reconceptualizing the ‘Anthropos’ in the Anthropocene: Integrating the Social Sciences and Humanities in Global Environmental Change Research.” Environmental Science & Policy 28: 3–13. 10.1016/j.envsci.2012.11.004.

[R35] ReActTube . 2017. “‘Antibiotic Resistance: The Silent Tsunami.’ ReActTube.” https://www.youtube.com/watch?v=LX6XHvFdzeY.

[R26] Regeringskansliet . 2016. “Svensk Strategi Förarbetet Mot Antibiotikaresistenst.” Stockholm: Regeringskansliet.” http://www.regeringen.se/4a8234/contentassets/7b70f26ea0e74e18ab6cd1cc5d3f030f/svensk-strategi-for-arbetet-mot-antibiotikaresistens.pdf.

[R30] Scott J. W . 2011. The Fantasy of Feminist History. Durham: Duke University Press. 10.2307/j.ctv1220mqq.

[R27] Silver L. L . 2011. “Challenges of Antibacterial Discovery.” Clinical Microbiology Reviews 24 (1): 71–109. 10.1128/CMR.00030-10. 21233508 PMC3021209

[R28] Sjöstedt Landén A . 2011. “Exploring Ideological Fantasies on the Move.” In Tracking Discourses: Politics, Identity and Social Change, edited by Sjölander Annika Egan and Gunnarsson Payne . Lund: Nordic Academic Press.

[R29] Uppsala University . 2017. “Antibiotic Resistance: The Silent Tsunami, Future Learn, MOOC.” Uppsala: Uppsala University. https://www.mooc-list.com/course/antibiotic-resistance-silent-tsunami-futurelearn.

[R31] WHO . 2014. “‘Antimicrobial Resistance: Global Report on Surveillance.’ France: WHO.” https://apps.who.int/iris/bitstream/handle/10665/112642/9789241564748_eng.pdf?sequence=1.

[R32] WHO . 2017. “Antibiotic Resistance. Fact Sheet.’ Updated November. France: WHO.” http://www.who.int/mediacentre/factsheets/antibiotic-resistance/en/.

